# The role of emotion in eating behavior and decisions

**DOI:** 10.3389/fpsyg.2023.1265074

**Published:** 2023-12-07

**Authors:** Oh-Ryeong Ha, Seung-Lark Lim

**Affiliations:** Department of Psychology, University of Missouri – Kansas City, Kansas City, MO, United States

**Keywords:** emotion, negative emotions, emotional eating, hedonic eating, eating behavior, reward values, eating decisions

## Abstract

The present paper aims to provide the latest perspectives and future directions on the association between emotions and eating behavior. We discussed individual differences in the impact of negative emotions on eating, emotional eating as disinhibited eating decisions with heightened reward values of and sensitivity to palatable foods in response to negative emotions and social isolation, in addition to emotional eating as maladaptive coping strategies under negative emotion and stress, hedonic (pleasure-oriented) eating decisions mediated by the brain reward system, and self-controlled (health-oriented) eating decisions mediated by the brain control system. Perspectives on future directions were addressed, including the development of early eating phenotypes in infancy, shared neural mechanisms mediated by the ventromedial prefrontal cortex and the dorsolateral prefrontal cortex in emotion and eating decision regulation, possible roles of interoception incorporating hunger and satiety signals, gut microbiome, the insula and the orbitofrontal cortex, and emotional processing capacities in hedonic eating and weight gain.

## Introduction

Emotions affect human behavior in various ways, such as motivating goal-directed behaviors, anticipating future outcomes, and supporting reward learning (Dolan, 2002). Emotions also can influence our food consumption and eating decisions regarding how much and when to eat certain foods (Meule and Vogele, 2013). Research has shown that the interplays across different types and intensity of emotions (e.g., negative vs. positive), eating styles (e.g., emotional eating—a tendency to eat in response to negative emotions; restrained eating—cognitive and behavioral restriction of food intake to control body weight; external eating—high susceptibility to food cues that results in craving for high-caloric, palatable foods), weight status, and types of foods (e.g., energy-dense, low-nutritious, unhealthier foods vs. nutritious, healthier foods) lead to different eating behaviors. Yet, emotion-eating mechanisms have not been fully identified. The mechanisms underneath emotion-related changes in eating behavior are multifaceted due to high variabilities across individuals and emotions (Macht, 2008). We focused on current findings exploring the impact of negative emotions on eating behavior and the decision-making mechanism in emotional eating, which provided perspectives on future research directions.

## Emotions and their impacts on eating behavior

More often than not, negative emotions tend to decrease food consumption, while positive emotions increase it (Evers et al., 2018; Reichenberger et al., 2018). However, negative emotions can evoke external eating in high-emotional eaters than in low-emotional eaters (Blechert et al., 2014). Negative emotions can increase food consumption in restrained eaters as well due to disturbed cognitive inhibition of food intake (Evers et al., 2018). Furthermore, negative emotions do not necessarily induce increased eating among emotional eaters or individuals with overweight or obese (Evers et al., 2018; Zhou and Tse, 2020).

To explain the inconsistency and varying impacts of negative emotions on eating, recent studies have proposed the importance of individual differences including individualistic emotion-eating experiences and the impact of discrete emotions (Altheimer and Urry, 2019). They emphasized a learned association between discrete negative emotions and eating, rather than general negative emotions, which can vary across individuals (Altheimer and Urry, 2019). People who have previously formed an association between emotion and eating (food consumption or restriction) are more likely to engage in emotional or restricted eating in response to discrete negative emotions like depression and anxiety. For example, sadness increased food consumption more than joy in women high-emotional eaters, while sadness and joy did not differently influence food intake in low-emotional eaters (van Strien et al., 2013). Moreover, depression was related to weight gain regarding body mass index (BMI) change in only women emotional eaters, but not in men emotional eaters as well as external eaters and restricted eaters in either sex (van Strien et al., 2016).

Recent research has elucidated individual differences in how emotions impact hedonic eating (i.e., taste-oriented intake of high-caloric and low-nutrient food with high sugar, salt, and fat for pleasure without hunger) and homeostatic eating (i.e., food intake with a hunger for regulating energy balance) in different contexts (Lutter and Nestler, 2009; Reichenberger et al., 2018; Devonport et al., 2019). Induced anger, fear, and sadness increased consumption of sweet foods (Salerno et al., 2014). But when sensitivity to long-term risks of hedonic eating was primed, sadness restricted hedonic eating, which may be relevant to the function of sadness to become more vigilant to a loss or harm (Salerno et al., 2014).

Both adults’ depression and emotional eating were related to hedonic eating and a higher BMI (Konttinen et al., 2010). More severe depressive symptoms were linked to less consumption of fruits and vegetables and more consumption of non-sweet energy-dense foods, but not was not related to more consumption of sweet foods when emotional eating was controlled. When depression was controlled, higher emotional eating was linked to more consumption of sweet and non-sweet energy-dense foods. Results demonstrate that experiencing sadness or depressive moods does not necessarily decrease energy intake. Negative emotions are likely to increase overeating of energy-dense foods and decrease consumption of healthier options. Furthermore, the interaction between negative emotions and emotional eating matters. When emotional eating was examined using ecological momentary assessments (EMA) where people reported their emotions and eating behavior for 10 days, emotional eating and BMI influenced hedonic and homeostatic eating (Reichenberger et al., 2018). High negative emotions increased hedonic eating in high-emotional eaters but decreased it in low-emotional eaters; high negative emotions increased homeostatic eating in people with higher BMI. In addition, emotional processing capabilities may be linked to emotional eating in individuals with obesity. Similar to individuals with anxiety disorders or depression who often demonstrate difficulties in recognizing negative emotions (Demenescu et al., 2010), individuals with obesity are more likely to show difficulties in recognizing fearful and angry facial expressions (Scarpina et al., 2021) and describing and regulating emotions (Fernandes et al., 2018). Challenges in emotional processing may reflect emotional avoidance to cope with negative emotions related to body dissatisfaction or weight stigmatization in individuals with obesity (Fernandes et al., 2018), which could lead to emotional eating.

Findings suggest that emotional eating is prone to unhealthier, taste-oriented food choices under negative emotions. Negative emotions tend to focus on short-term gratification at the expense of long-term health risk considerations. Given the relationship between emotional eating and less adaptive coping strategies like emotion-oriented (i.e., coping through regulating negative emotional responses) and avoidant coping (i.e., avoiding or distracting from negative stress responses) (Spoor et al., 2007), hedonic eating could reflect less adaptive coping attempts for mitigating negative emotions in emotional eaters (Goossens et al., 2009).

## Food-evoked emotions, reward values, and food decisions

Food consumption is most likely to elicit positive emotions like satisfaction and enjoyment rather than negative emotions (Desmet and Schifferstein, 2008). Conceptually, people associate sweet taste with positive emotion-laden words like happiness, love, grateful, acceptance, hug, and kiss, and bitter taste with negative emotion-laden words like sad, disgust, and rejection (Zhou and Tse, 2020). Sensory experiences can be embodied in psychological states and emotions through repeated associations between taste and emotion (e.g., associations between sweet taste and happiness) from early on in our lives (Zhou and Tse, 2020, 2022).

Individualistic food experiences from prenatal periods may impact sensory preferences. Infants are predisposed to like sweet and salty tastes and dislike sour and bitter tastes (Birch and Fisher, 1998). It has adaptive values that allow infants to find safe food sources like the sweet taste of breast milk. However, in the most modern obesogenic societies of food abundance, a preference for sweet, salty, and fatty foods is more likely to result in poorer nutritious choices and weight gain (Birch and Fisher, 1998). Developing less predisposed-taste-oriented food choices can be critical for physical and mental health. Accepting more nutritious foods (e.g., leafy vegetables and beans) and learning to regulate emotions adaptively could prevent disinhibited overconsumption of sweet, high-caloric, low-nutritious foods in response to negative emotions and stress. Nevertheless, challenges lie in that we are inclined to “eat” palatable and energy-dense foods rather than reject foods for secure energy intake (Ha et al., 2016). Anticipating and consuming palatable foods are associated with pleasure mediated by the brain reward system (Kringelbach et al., 2012; Berridge and Kringelbach, 2013; Volkow et al., 2013; Alonso-Alonso et al., 2015). Moreover, food taste preference is a major determinant of eating decisions (Raghunathan et al., 2006; Gutjar et al., 2015; Ha et al., 2019).

People make eating decisions based on subjective reward values of food attributes, specifically, food taste and health. Decision values of food taste and health attributes are encoded in the ventromedial prefrontal cortex (vmPFC) that involved in reward value computation (Hare et al., 2009; Clithero and Rangel, 2013; Bruce et al., 2016; Lim et al., 2023). Hedonic, pleasure-oriented decisions that predominantly incorporate food taste attributes lead to less nutritious, unhealthier eating and/or weight gain (Lim et al., 2018, 2023). To make more nutritious, healthier decisions, the health attributes of food need to be considered. In health-oriented, self-controlled decisions (i.e., refusing tasty but unhealthy foods or eating not-tasty but healthy foods) mediated by the brain control system (Chib et al., 2009; Hare et al., 2009; Lim et al., 2016, 2023), food health attributes are incorporated early and significant enough during decision process (Sullivan et al., 2015; Lim et al., 2018), which allows people to pursue long-term health benefits rather than immediate pleasure from palatable, but less nutritious and unhealthier foods (Ha et al., 2016, 2021; van Meer et al., 2016; Lim et al., 2018).

Emotional eating is related to impulsive, less self-controlled decisions in response to negative emotions or stress (Elfhag and Morey, 2008; Verstuyf et al., 2013; Zhu et al., 2014). Emotional eating tends to be associated with external eating (e.g., overconsumption in response to external food cues like the smell of food and food advertisements) (van Bloemendaal et al., 2015; van Strien, 2018), which supports lapses of self-control in emotional eating. Overriding immediate pleasure for long-term health goals becomes arduous for emotional eaters when emotional distress increases reward values of palatable, high-caloric foods and sensitivity to those food cues in the brain reward system including the orbitofrontal cortex (OFC) and ventral striatum (Wagner et al., 2012; van Bloemendaal et al., 2015). Under negative emotions or stress, heightened sensitivity to food cues and reward values lead to increased cravings for palatable foods and disinhibited eating in people with obesity as well (Jastreboff et al., 2013). Craving, or motivations for seeking palatable foods are encoded in the mesolimbic dopamine reward system, especially in the substantia nigra (SN) and ventral tegmental area (VTA) (Meye and Adan, 2014). Intranasal oxytocin administration reduces the consumption of palatable foods and decreases activations in the VTA in response to high-calorie food cues in men with overweight and obesity (Plessow et al., 2018). These findings suggest that associations between negative emotion and hedonic eating are linked to changes in the brain reward system, which make people more prone to craving and overconsumption of palatable foods, especially among emotional eaters and people with excessive weight.

Hedonic eating is also linked to affection and social needs. Sweet taste often forms an association with warmth and love (Chan et al., 2013; Ren et al., 2015). Sweet taste and a romantic partner activate similar neural responses in the anterior cingulate cortex that are involved in the reward system (Bartels and Zeki, 2000; Araujo et al., 2003). When people are lacking warmth and love, deprivation of positive emotions and social connection could result in compensatory reward-seeking behaviors, including craving and over-consumption of low-nutritious sweet foods (Henriksen et al., 2014; Tomova et al., 2020; Doan et al., 2022). Loneliness increased craving for sugar-sweetened beverages measured using an EMA in adolescents (Doan et al., 2022). Loneliness increased the consumption of sweet beverages, while perceived social connection decreased the consumption of sweet beverages in pregnant women (Henriksen et al., 2014). In young adults, deprivation of social connection and food evoked similar behavioral and neural responses (Tomova et al., 2020). Acute social isolation resulted in increased negative emotions and increased social craving, and fasting resulted in increased negative emotions and increased food craving and hunger (Tomova et al., 2020). The level of self-reported cravings for social connection and cravings for food after deprivation were strongly correlated. Further, both types of deprivation evoked similar responses in dopaminergic midbrain regions involved in craving and seeking palatable foods, specifically in the SN and VTA.

In sum, food evokes positive emotions. Learned associations between food and negative emotions may heighten emotional eating that is prone to disinhibited eating decisions of seeking palatable foods in response to negative emotions, social isolation, and stress. For better mental and physical health, it will be crucial to develop more adaptive emotion regulation, coping strategies, and health-oriented, self-controlled eating decisions.

## Discussion

While much is yet to unfold, recent advances may shed light on new perspectives in understanding the relationship between emotion and eating behaviors.

Exploring pleasure-seeking and eating phenotypes in early life may provide important knowledge about the development of hedonic eating. Food preferences and appetite traits begin to develop from prenatal periods influenced by genetic predispositions and maternal food choices (Ventura and Worobey, 2013). Given that infants transition to adult foods during the first 2 years (Carruth et al., 2004), early food experiences could lay the foundation for food preferences (Domel et al., 1996; Gibson et al., 1998; Howard et al., 2012; Mura Paroche et al., 2017). One of the prominent obesogenic eating phenotypes is sensitivity to external food cues, which often results in overeating that potentially leads to obesity (Carnell et al., 2013). One study demonstrated that 6- to 12-month-olds with rapid weight gain showed higher responsivity (touching) to foods over nonfoods, suggesting that early heightened sensitivity to food cues could influence rapid weight gain (Buvinger et al., 2017). Moreover, 9- to 18-month-old infants with more rapid weight gain (i.e., greater weight-for-length z scores) found a favorite food was more rewarding than nonfood alternatives (a toy and DVD) compared to lean infants. Interestingly, reward values of nonfood alternatives were significantly lower among infants with more rapid weight gain than lean infants. These findings suggest that infants with rapid weight gain have developed heightened food reward-seeking along with a lack of alternative sources for pleasure-seeking, which could contribute to hedonic eating and weight gain in their later lives. It will be worth examining how early preferences to food and other objects are associated with the development of hedonic eating and risks for obesity.

A more holistic understanding of shared neural mechanisms of emotion and eating behavior may shed light on the intervention of hedonic eating. Common neural mechanisms of emotion and eating behavior regulations have been identified (Godet et al., 2022). The vmPFC mediates valuations of emotional food stimuli considering people’s current goals and contexts (Ochsner et al., 2012; Lim et al., 2023). The dorsolateral prefrontal cortex (dlPFC) mediates both emotion regulations (Ochsner and Gross, 2005; Ochsner et al., 2012) and eating regulations (Hare et al., 2009; Hutcherson et al., 2012) by incorporating long-term goals into value representation in the vmPFC (Hare et al., 2009; Hutcherson et al., 2012). Recent research has demonstrated the positive effect of the dlPFC modification using transcranial direct-current stimulation (tDCS) on emotion, such as reduced emotional reactivity to negative emotions (Clarke et al., 2020), enhanced cognitive control of emotion regulation (Feeser et al., 2014), and decreased anxiety and stress responses measured by cortisol levels (Mehrsafar et al., 2020). Similarly, some research demonstrated that the dlPFC modulation using tDCS reduced food craving, liking, and/or consumption in healthy-weight adults with higher food craving (Jauch-Chara et al., 2014; Kekic et al., 2014; Lapenta et al., 2014; Anderson et al., 2023), in adults with overweight and higher food craving (Ljubisavljevic et al., 2016), and adults with obesity and binge eating disorder (Burgess et al., 2016). However, other studies did not find significant changes in food cravings, choice, and consumption (Georgii et al., 2017; Ray et al., 2017; Beaumont et al., 2021). Individual differences in eating and psychological aspects could play a role in inconsistent findings, given results vary depending on the level of susceptibility to hedonic eating, types of eating behaviors, and psychological traits (Ray et al., 2017). For example, the dlPFC modulation via tDCS did not change food cravings and consumption in healthy-weight adults with lower hedonic eating (Beaumont et al., 2021). The dlPFC modulation reduced food liking (pleasure) but not unhealthy food choices in healthy-weight adults with higher food cravings (Anderson et al., 2023). Further research is necessary to determine the effectiveness of neural modulation in food craving and consumption.

Recently, individual differences in interoception and gut microbiome have been considered to provide an insight of the association between emotion and eating. Interoception is the ability to accurately sense internal body signals (Garfinkel et al., 2015; Khalsa et al., 2018). Poorer interoception is linked to worse emotion regulation (Füstös et al., 2012), higher emotional eating and BMI (Robinson et al., 2021), and disordered eating (Ahlich and Rancourt, 2022). Hypersensitivity to interoceptive hunger signals and less accurate detection of satiety and energy balance signals are prone to hedonic eating and obesity (Simmons and DeVille, 2017). The variation in gut microbiome consisted of 10^13^ to 10^14^ microorganisms inhabiting the gastrointestinal system (Le Chatelier et al., 2013) including reduced bacterial diversity and altered composition may contribute to delayed satiety setpoint (Yatsunenko et al., 2012), food craving (Alcock et al., 2014), dysregulated energy balance and weight gain (Le Chatelier et al., 2013; Davis, 2016; Torres-Fuentes et al., 2017), and disordered eating (Terry et al., 2022). Via the vagus nerve, some gut microbiota and gastrointestinal signals are delivered to the brain, which bidirectionally influence emotional experiences like anxiety and stress (Breit et al., 2018). The insula integrates projected interoceptive signals with emotional, cognitive, and motivational signals that result in explicit experiences of positive and negative emotions (Namkung et al., 2017). The anterior insula relays saliency signals associated with emotions (e.g., pleasure, pain) to dlPFC for initiating attentive control (Menon and Uddin, 2010) and represents past emotional experiences in similar contexts to vmPFC to evaluate outcomes for future decisions (Namkung et al., 2017). The anterior insula is a part of the primary taste and olfactory cortex (Rolls, 2016), along with the OFC involved in the modulation of taste reactions, affective representation of rewards (e.g., pleasure from palatable foods), and food-evoked emotion (Kringelbach, 2005). Research reported that higher emotional eating was linked to greater activations of the insula and the OFC (Bohon et al., 2009; van Bloemendaal et al., 2015) but see Bohon (2014). These findings may account for the multifaceted role of the insula and the OFC across interoception, emotion generation and modulation, taste perception, and responses to food cues, which suggests overlapping mechanisms under emotion and food processing (Frank et al., 2013).

Altogether, we have addressed commonalities across emotion and eating, while emphasizing the importance of variability and individual differences in understanding the association between emotion and eating behaviors. Further investigations on neural, physiological, and decision-making mechanisms of emotional eating and effective intervention and prevention of hedonic and emotional eating are warranted.

## Author contributions

O-RH: Writing – original draft, Writing – review & editing. S-LL: Writing – review & editing.
